# The Cutting Edge Geometric Optimization of the PCBN Tool for the Machining of Cast Iron

**DOI:** 10.3390/mi16090978

**Published:** 2025-08-26

**Authors:** Xian Wu, Zhiqin Su, Chao Zhang, Xuefeng Zhao, Hongfei Yao, Feng Jiang

**Affiliations:** 1 School of Mechanical Engineering, Guizhou University, Guiyang 550025, China; zxf801112@163.com; 2 Zhejiang Xinxing Tools Co., Ltd., Jiaxing 314300, China; 22014080109@stu.hqu.edu.cn (C.Z.); yhf@ch-tools.com (H.Y.); 3 College of Mechanical Engineering and Automation, Huaqiao University, Xiamen 361021, China; szhq1997@163.com (Z.S.); jiangfeng@hqu.edu.cn (F.J.)

**Keywords:** tool design, edge geometric, PCBN tool, cutting simulation

## Abstract

The turning process is the main machining task in brake disc production, and the PCBN tool is the most suitable type of cutting tools in the machining of brake discs made of cast iron. The edge geometric optimization of the PCBN tool is the key factor to obtain a better tool performance. In this paper, the cutting simulation for the machining of cast iron with PCBN tool of grade HNMN120712 was established, which exhibits a simulation error lower than 10.8%. The optimal turning parameters were obtained by the equal material removal rate method. The edge geometric parameters were optimized in two stages: firstly, the optimal edge radius was obtained as 30 μm by the comprehensive normalization analysis of the cutting temperature and stress, and then, the chamfer width and angle were further optimized to 0.1 mm and 15°. At finally, the optimized PCBN tool was prepared and tested in the machining of brake discs; the results indicate that the designed tool exhibits an excellent tool performance with 3.4 times the tool life of the conventional tool.

## 1. Introduction

Cast iron has been widely applied to produce brake discs in the vehicle field because of its significant advantages, such as good intensity, better thermal conductivity, excellent wear resistance, and low price [1,2]. The production process of brake discs usually includes the casting process and the subsequent mechanical machining process. The employed process in the machining of brake discs includes the turning of the outer circle and end face, the groove milling on the end face, and drilling. In these operations, the turning process is the main operation and occupies the most considerable machining task and longest time. The cutting tool has a great effect on the machining quality in the turning of brake discs. Among the different cutting tools, the polycrystalline cubic boron nitride (PCBN) tool is the most suitable type of tool in the machining of cast iron. The optimization design of the PCBN tool structure is key to obtain better machining quality [3,4,5].

Different machining conditions usually need different tool structures, such as different workpiece materials, different machining parameters, and rough or fine machining process [6,7]. The geometric parameters of the PCBN tool for the turning process have a significant effect on the tool performance, such as the rake and flank angles, tool tip radius, tool chamfer, and edge radius. Among these parameters, edge geometric parameters are the most important [8,9]. Chen [10] proposed the variable chamfer edge of PCBN tools, and found it exhibits obvious advantages to decrease tool wear and improve tool life, by comparing to the uniform chamfer edge. Li [11] compared the sine-strengthened edge and negative chamfer edge of PCBN tools, and found that the sine-strengthened edge can obtain a better tool performance. Chen [12] found that the cosine-enhanced PCBN tool exhibits a better tool performance, and further proposed the double-inclined-wall cosine-enhanced edge, which has a lower tool wear. Zhang [13] studied wear mechanisms of PCBN tools with different edge radii in cutting of Zr-based bulk metallic glasses, and found the medium edge radius obtains the best machining quality.

The reasonable tool structure directly determines the cutting performance and processing quality for the corresponding conditions [14,15]. The cutting simulation is an effective method for tool design and mechanism study, and is widely used in many fields. Hussain [16] studied the effect of tool geometric parameters and machining conditions by cutting simulation, and found it is effective and efficient to predict the cutting performance. Agmell [17] used a numerical and experimental method to study the mechanism in machining of Inconel 718 with a PCBN tool, and found the simulation was well correlated with the experimental results. Yang [18] used the cutting simulation to optimize the rake angle of a micro-drill with a better tool performance. Liao [19,20] obtained the optimal geometrical parameters of tool chip breaker and the cutting edge by cutting simulation. Lei [21] obtained the optimal tool geometry design and preferred processing parameters of micro flat-end milling to machine soft-brittle material with cutting simulation.

To obtain the optimal tool geometric parameters of a PCBN tool for the machining of brake discs, the cutting simulation model of cast iron materials was established. Firstly, the cutting parameters for the machining of cast iron materials were optimized based on the equal material removal rate. And then, the cutting geometric parameters, including the cutting edge radius and chamfer parameters, were further optimized with the cutting simulation. Finally, the optimized PCBN tool was verified in the machining of brake disc parts. The results can offer an important guideline for the tool design and selection in the machining of brake discs.

## 2. The Cutting Simulation of Cast Iron Material

### 2.1. The Constitutive Model of Cast Iron Material

**Figure 1:**
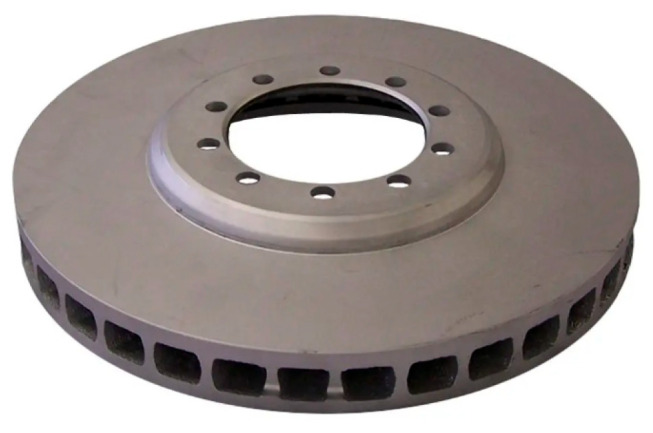
The cast iron FC220P for brake discs.

The brake disc is a key part in the braking system of various cars. Gray cast iron has always been a commonly used material for brake discs, which present high strength, good thermal conductivity, and wear resistance. Among different cast iron materials, the material with grade of FC220P is widely used for brake discs in the major brands of Japanese cars, as depicted in Figure 1. The common mechanical properties of cast iron FC220P are depicted in Table 1.

The constitutive model of workpiece material is a significant factor to obtain a good simulation precision. Constitutive models proposed in previous research include the Johnson–Cook (J-C) model and the Power–Law (P-L) model. In this work, the P-L constitutive model was adopted, and the expression of the P-L constitutive model is depicted as follows [22]:

(1) $$\sigma (\varepsilon_{s},{\dot{\varepsilon }}_{s},T)=g(\varepsilon_{s})\cdot \Gamma ({\dot{\varepsilon }}_{s})\cdot \Theta (T)$$

(2) $$g(\varepsilon_{s})=\sigma_{0}{\left(1+\frac{\varepsilon_{s}}{\varepsilon_{0}}\right)}^{\frac{1}{n}}$$

(3) $$\Gamma ({\dot{\varepsilon }}_{s})={\left(1+\frac{{\dot{\varepsilon }}_{s}}{{\dot{\varepsilon }}_{0}}\right)}^{\frac{1}{m}}$$

(4) $$\Theta (T)=C_{0}+C_{1}T+C_{2}T^{2}+C_{3}T^{3}+C_{4}T^{4}+C_{5}T^{5}$$

According to the above equation, the P-L constitutive model includes three parts, the strain hardening term $g(\varepsilon_{s})$, the strain rate strengthening term $\Gamma ({\dot{\varepsilon }}_{s})$, and the thermal softening term Θ(T), which are respectively expressed in Equations (2)–(4). In these equations, *σ*_0_ is reference yield strength, *ε*_0_ is reference strain, $\varepsilon_{s}$ is strain, *n* is strain hardening coefficient, ${\dot{\varepsilon }}_{0}$ is reference strain rate, ${\dot{\varepsilon }}_{s}$ is strain rate, *m* is strain rate strengthening coefficient, *T* is temperature, and *C*_0_, *C*_1_, …, *C*_5_ is the coefficient of the thermal softening term.

**Figure 2:**
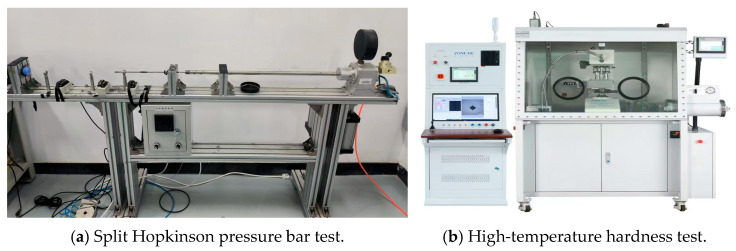
Constitutive model test of the cast iron material.

Due to the high strain and strain rate of the workpiece material in the cutting process, the constitutive model test for the cutting simulation requires higher strain rates. The split Hopkinson pressure bar test can obtain strain rates of 10^2^~10^4^/s^−1^, which are suitable to obtain the P-L constitutive model parameters of cast iron FC220P for the cutting simulation. In this work, the P-L constitutive model of cast iron F220P was established by the split Hopkinson pressure bar test and high-temperature hardness test, as depicted in Figure 2. According to previous research [23], the P-L constitutive model of cast iron was calculated as shown in Equation (5):

(5) $$\begin{array}{c} \sigma (\varepsilon_{s},{\dot{\varepsilon }}_{s},T)=525.35\times (1+\frac{\varepsilon_{s}}{0.0053}{)}^{0.1196}\times (1+\frac{{\dot{\varepsilon }}_{s}}{1050}{)}^{0.0861}\\ \times \;(0.9932+3.005e^{-4}T-1.314e^{-6}T^{2}+7.737e^{-10}T^{3}) \end{array}$$

### 2.2. The Cutting Simulation Model of PCBN Tool

**Figure 3:**
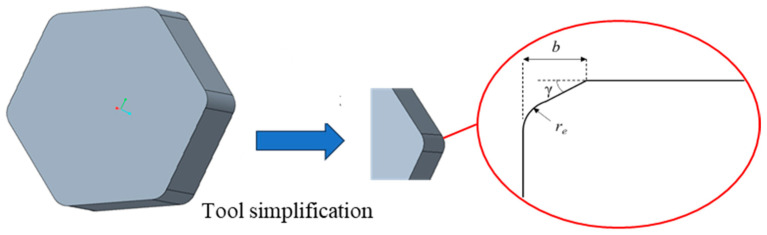
The cutting edge geometric parameters of the PCBN tool.

Although there are many different tool types for the turning of brake discs, such as tool types with the shapes of rhombus, square, and regular hexagon (codes C, D, S, and H), in comparison, the tool type with the shape of a regular hexagon presents the maximum quantity of six cutting edges and has the greatest cost-effectiveness. Hence, the tool grade of HNMN120712 was included in the PCBN tool for the machining of brake discs, which presents the rake and flank angles of 0°, tool tip wedge angle of 120°, and tool tip radius of 1.2 mm. The cutting edge geometric parameters are the most significant parameters for the PCBN tool with the grade of HNMN120712, which greatly affect the machining quality and tool life. However, there is still no existing literature about the cutting edge geometric optimization of the PCBN tool with the grade of HNMN120712. Hence, the cutting edge geometric optimization of the PCBN tool with the grade of HNMN120712 was performed in this work, as depicted in Figure 3. In the cutting simulation, the PCBN tool was simplified to one cutting edge, and *r_e_* is the cutting edge radius, γ is the chamfer angle, and *b* is the chamfer width.

**Figure 4:**
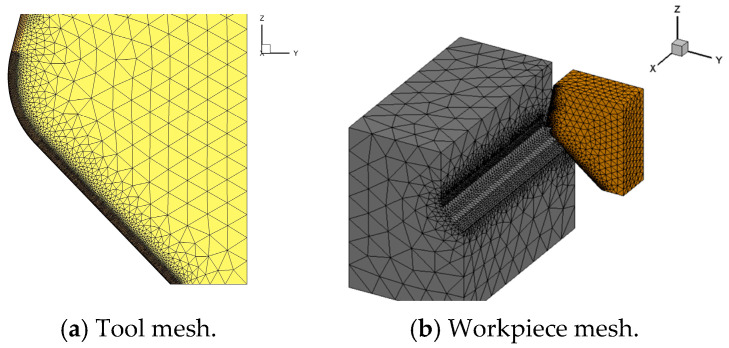
The cutting simulation model of the PCBN tool.

In this work, the cutting simulation was performed by the software AdvantEdge 7.1. The turning process was simplified to the straight cutting process, as depicted in Figure 4. The PCBN tool was set to a rigid body, and the size of the cast iron workpiece was set to 15 × 8 × 6 mm; the P-L constitutive model was set according to the obtained Equation (5). The original temperature was set to 25 °C, and the heat conduction between the tool and workpiece was mainly considered. The tool–chip friction coefficient was set to 0.3 based on the cutting experiment results [23]. The mesh division level of the PCBN tool was set to 0.5, the maximum element sizes were 0.3 mm, and the minimum element was 0.003 mm. During the meshing of workpiece, the mesh refinement degree in the cutting zone was set to 0.018, and the mesh refinement factor was set to 10; the grid coarsening factor was set to 18. The cutting temperature and stress were averaged in the stable stage, with a cutting length range from 5 mm to 10 mm.

### 2.3. The Experiment Verification of Cutting Simulation

**Figure 5:**
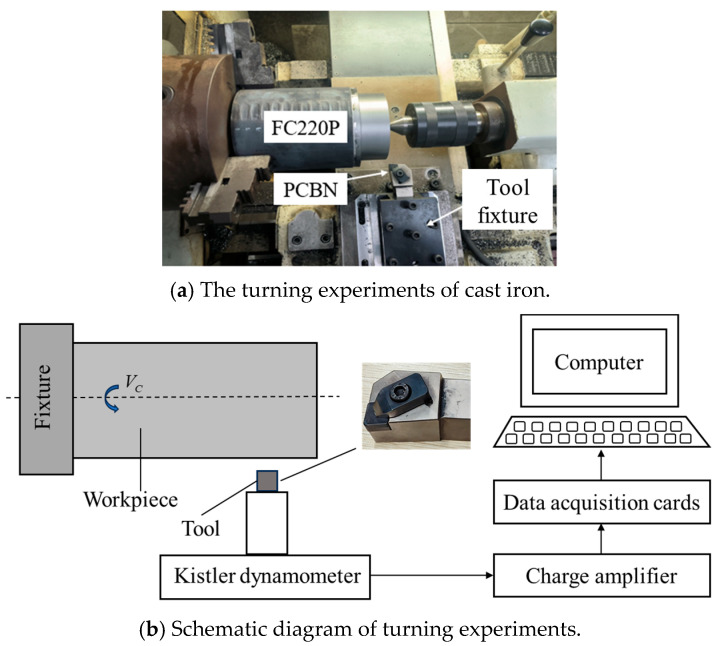
The turning experiments for cast iron.

The turning experiments were performed with the turning machine CAK6150 prepared by Shenyang Machine Tool Co., Ltd. (Shenyang, China), as depicted in Figure 5. This machine can provide the largest spindle rotation of 2200 rpm and a machining accuracy of ±8 μm. The cutting tool in the experiment was the PCBN tool with the grade of HNMN120712, the same as in the cutting simulation, and the workpiece in the experiment was the cast iron F220P with a size of Φ140 × 200 mm. In order to be consistent with the cutting simulation, the drying cutting condition was performed. To verify the cutting simulation results, the cutting force was acquired by the dynamometer of Kistler 9257B (Kistler, Winterthur, Switzerland). The measurement range of the dynamometer was ±5 kN, which fully met the force measurement requirements. The sampling frequency was set to 20,000 Hz in the cutting force measurement.

By considering the production efficiency requirement of brake discs in the actual workshop, the turning parameters were set according to the equal material removal rate method. As depicted in Table 2, the cutting depth *a_p_* was set to the constant value of 1 mm, and the feed rate *f* and the cutting velocity *v* were synchronously changed to obtain the equal material removal rate of 2.5 × 10^5^ mm^3^/min. The turning experiments were repeated three times, and the averaged cutting force was adopted as the results.

**Figure 6:**
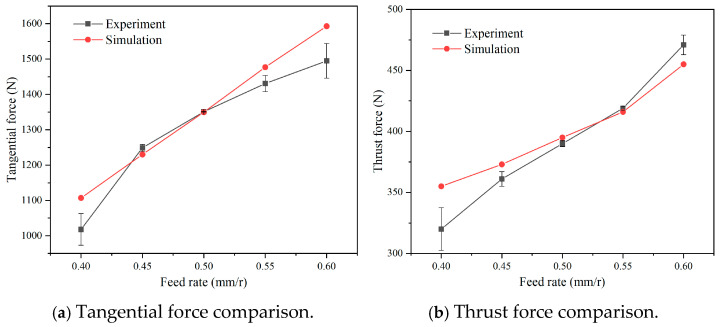
The experimental verification of the cutting simulation.

The acquired cutting force from the turning experiments and the simulated cutting force are depicted in Figure 6. It was found that, under an equal material removal rate, both the tangential force and thrust force gradually raise with the increase in the feed rate and the decrease in the cutting velocity. The experimental and simulated tangential forces rise from 1018 N and 1107 N to 1495 N and 1593 N, respectively. The thrust forces increase from 320 N and 355 N to 471 N and 455 N for the experimental and simulated results, respectively. According to the results, the experimental cutting forces are almost consistent with the simulated cutting forces. By comparing the experimental and simulated cutting forces, the largest error in the tangential force is 8.7%, and the largest error in the thrust force is 10.8%, which is actually in the acceptable error range for the cutting simulation. This result verifies the accuracy and precision of the established cutting simulation, which is subsequently used to optimize the cutting edge geometric parameters of the PCBN tool for the machining of cast iron.

## 3. The Cutting Parameters’ Optimization Based on Equal Material Removal Rate

**Figure 7:**
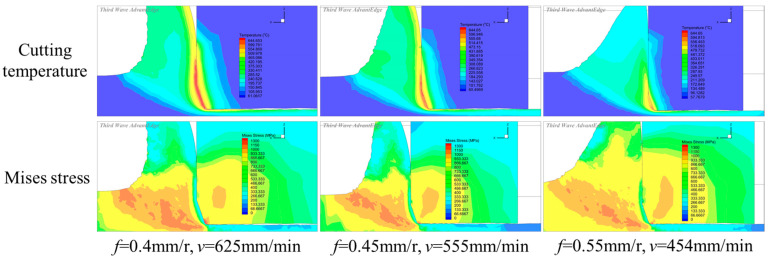
The cutting temperature and stress distribution under different parameters (*a_p_* = 1 mm).

From the turning experiments, it was found that, under an equal material removal rate, different turning parameters lead to different machining performances. To obtain the optimal turning parameters considering the equal production efficiency requirement, a cutting simulation was firstly performed to optimize the cutting parameters. The cutting parameters in the simulation were the same as those in Table 2; the cutting depth was fixed to *a_p_* = 1 mm, and the feed rate and cutting velocity were varied to obtain the same material removal rate. The simulated cutting temperature and stress distribution under different parameters are depicted in Figure 7. It was found that a high cutting temperature zone with a low feed and high velocity is mainly located on the chamfer and rake faces. With a large feed and low velocity, the high cutting temperature zone reduces the scope and is mainly located on the cutting edge roundness. The high stress zone with a low feed and high velocity is mainly located on the cutting edge roundness. With the increase in the feed rate, the high stress zone on the cutting edge roundness reduces the scope, and on the rake face increases the scope.

**Figure 8:**
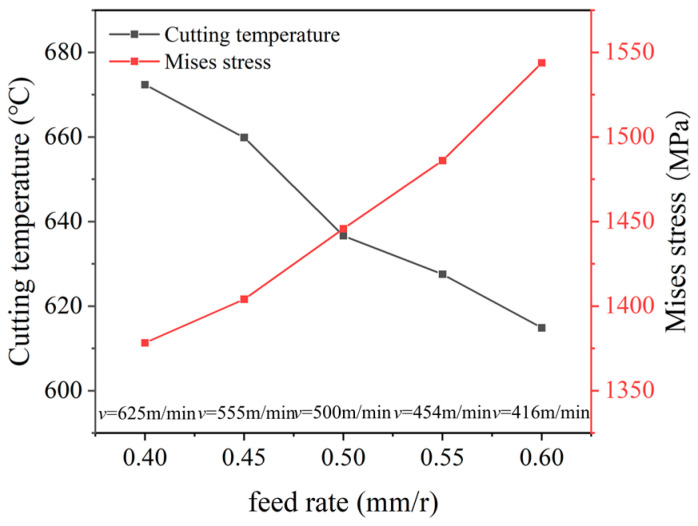
The cutting temperature and stress under different parameters (*a_p_* = 1 mm).

The statistic value of the cutting temperature and stress under different parameters are depicted in Figure 8. It was found that, under an equal material removal rate, when the feed rate increases from 0.4 mm/r to 0.6 mm/r, and the cutting velocity reduces from 625 m/min to 454 m/min, the temperature reduces from 671 °C to 613 °C, which exhibits a reduction ratio of 9.6%, but the stress increases gradually from 1380 MPa to 1547 MPa with an increase ratio of 10.7%. Under an equal material removal rate, the large feed rate can obtain a large cutting area but a low cutting velocity in the cutting process. The large cutting area causes an increased cutting force, and the low cutting velocity and the increased radiant area are helpful to reduce the cutting temperature.

The results indicate that, with the increase in the feed rate and reduction in the cutting velocity, the variation in the cutting temperature and stress is opposite; it is difficult to obtain the optimal parameters based on the single cutting temperature or stress. Hence, the data normalization $Q_{i}$ of the cutting temperature and stress was performed according to Equation (6), and then, their total was calculated with Equation (7) to obtain the optimal parameters.

(6) $$Q_{i}=(X_{i}-X_{min})/(X_{max}-X_{min})$$

(7) $$Q_{total}=\sum_{1}^{n}\left(Q_{i}\right)$$

**Figure 9:**
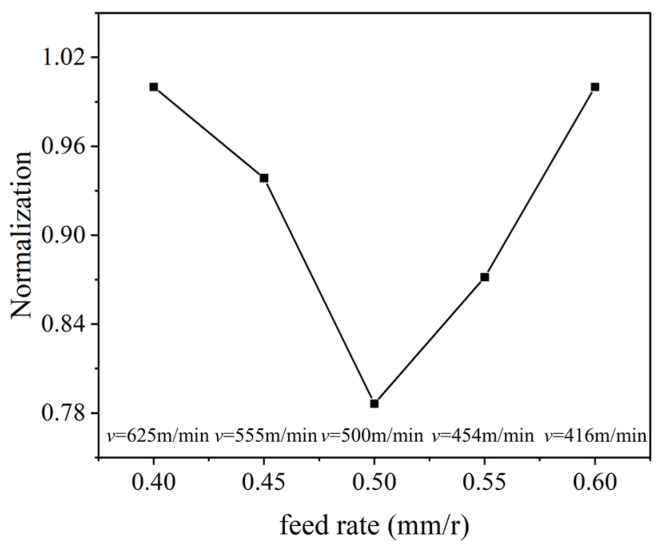
The cutting parameter optimization based on comprehensive normalization (*a_p_* = 1 mm).

The calculated comprehensive normalization value $Q_{total}$ of the cutting temperature and stress is depicted in Figure 9. It was found that the comprehensive normalization value firstly reduces and then increase with the increase in the feed rate; the minimum value was obtained with the feed rate of 0.5 mm/r, and the corresponding cutting speed was 500 m/min, which are the comprehensive optimal parameters under an equal material removal rate.

## 4. The Cutting Edge Geometric Optimization of PCBN Tool

The edge geometric parameters of the PCBN tool include the edge radius and the chamfer angle and width. The larger edge radius and chamfer parameters can enhance the edge strength, but the reduced sharpness of the cutting edge simultaneously may increase the cutting temperature and stress, and subsequently reduce the tool life. Hence, the edge geometric of PCBN tool was optimized in two stages: the edge radius was optimized with a single-factor simulation in the first stage, and then, the chamfer width and angle wee optimized with the full-factorial simulation in the second stage, as depicted in Table 3.

**Figure 10:**
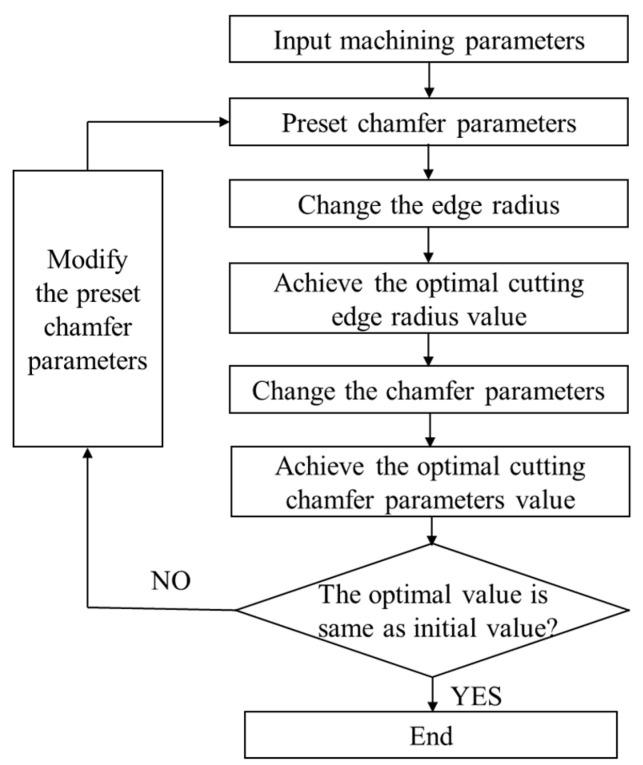
The optimization process of cutting edge geometric parameters.

The edge geometric of PCBN tool includes three variables to ensure effectiveness of the optimization. The edge geometric optimization process is depicted in Figure 10:

(1) Initialize the cutting simulation, and input the optimal machining parameters and the constitutive model of the cast iron FC220P and PCBN tool.
(2) Preset chamfer parameters, and perform the cutting simulation with different edge radii in the range from 10 μm to 50 μm, to obtain the comprehensive optimal edge radius based on the cutting simulation results.
(3) Based on the optimal edge radius, perform the cutting simulation with different chamfer parameters to obtain the comprehensive optimal chamfer parameters based on the cutting temperature and stress.
(4) If the optimal chamfer parameters are same to the preset value in step (2), end the optimization process. Otherwise, change the preset chamfer parameters in step (1), and then repeat the optimization process until the optimal chamfer parameters and the preset value are consistent.

### 4.1. The Cutting Edge Radius Optimization of PCBN Tool

**Figure 11:**
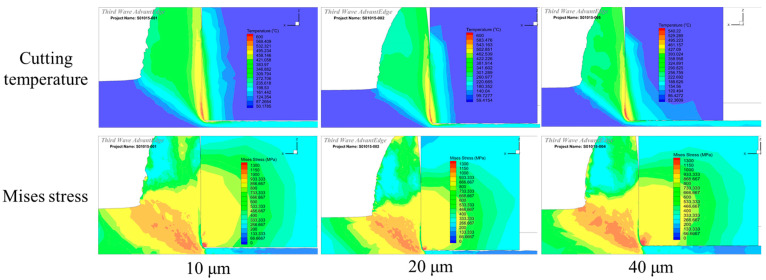
The cutting temperature and stress distribution under different edge radii (0.1 mm/15°).

In the first stage, according to the existing literatures [23], the chamfer width and angle were preset to two groups, which were 0.1 mm/15° and 0.15 mm/25°, and the influence of edge radius on the cutting temperature and stress was investigated with the cutting simulation. The cutting temperature and stress distribution under different edge radii are depicted in Figure 11, which are the main factors that affect the machining quality and tool life. It was found that, with an edge radius of 10 μm, the high cutting stress zone mainly concentrates on the small scope of edge roundness. The sharp edge can reduce the cutting deformation and cutting force in the cutting process, which is accompanied by a relatively lower cutting temperature on the chamfer face. However, the sharp edge simultaneously reduces the edge strength; instead, it results in a large cutting stress. On the contrary, when the edge radius increases to 40 μm, the cutting edge becomes blunt, and it increases the cutting deformation and the cutting force in the cutting process; this can be reflected by the high cutting temperature that concentrates on the chamfer face and edge roundness. Simultaneously, the blunt edge can increase the strength and stressed area in the cutting process, resulting in a relatively low value but large scope of the high cutting stress zone.

**Figure 12:**
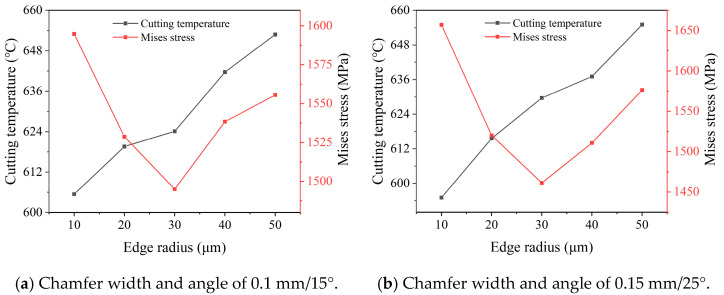
Effect of edge radius on the temperature and stress (*a_p_* = 1 mm, *f* = 0.5 mm/r, *v* = 500 m/min).

The counted value of the cutting temperature and stress under different radii are depicted in Figure 12. It was found that the variation in the cutting temperature and stress are the same under two groups of the preset chamfer parameters. The cutting temperature continuous rises with the increase in the edge radius. When the edge radius changes from 10 μm to 50 μm, it increases from 605 °C and 594 °C to 654 °C and 655 °C under different chamfer parameters, respectively. However, the cutting stress firstly reduces and then increases with the increase in the edge radius; the lowest cutting stress is achieved under the edge radius of 30 μm for both two groups of chamfer parameters.

**Figure 13:**
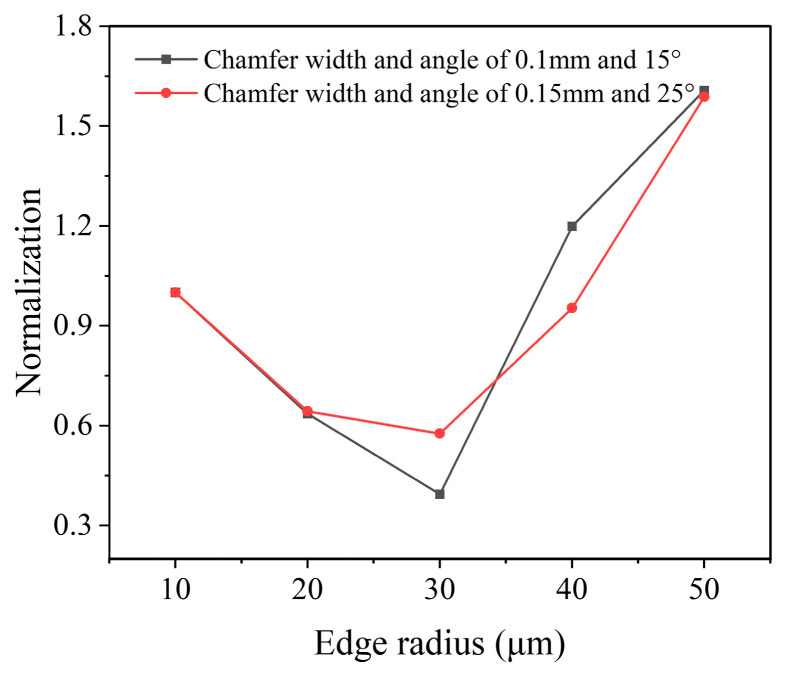
Edge radius optimization based on normalization (*a_p_* = 1 mm, *f* = 0.5 mm/r, *v* = 500 m/min).

To obtain the optimal edge radius that comprehensively considers both the cutting temperature and stress, data normalization with the results in Figure 12 was performed with Equations (6) and (7). The obtained comprehensive normalization value calculated for both chamfer parameters of 0.1 mm/15° and 0.15 mm/25° are depicted in Figure 13. According to the results, the smallest comprehensive normalization value is achieved with the edge radius of 30 μm, the same to the edge radius of the smallest cutting stress. The results indicate that the optimal edge radius for the PCBN tool is 30 μm, which leads to the best tool performance.

### 4.2. The Chamfer Parameters’ Optimization of PCBN Tool

**Figure 14:**
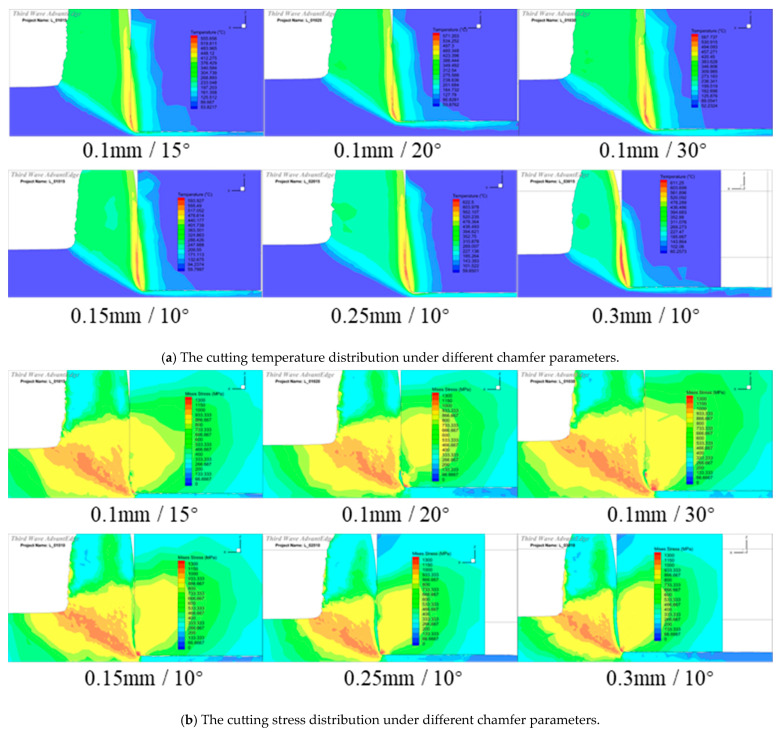
The temperature and stress distribution with different chamfer parameters (*r_e_* = 30 μm).

After optimizing the edge radius, the chamfer parameters were further optimized with the full-factorial simulation. In the simulation, five levels of the chamfer width were selected in range of 0.1~0.3 mm, and five levels of the chamfer angle were selected in the range of 10~30°, as depicted in Table 3. In the simulation, the cutting parameters were set to *a_p_* = 1 mm, *f* = 0.5 mm/r, and *v* = 500 m/min; the edge radius was set to 30 μm, based on the above results. The cutting temperature and stress under different chamfer parameters are depicted in Figure 14. The increase in the chamfer parameters can enhance the edge strength but also passivate the edge. When the chamfer angle increases from 10° to 30°, the rake angle on edge roundness actually becomes more negative, and the high cutting temperature zone on the chamfer face becomes more and more obvious. The highest cutting temperature is located on the intersection point of the chamfer face and rake face. However, the high cutting stress zone is located on the edge roundness, but not the chamfer face; it also exhibits a large scope with the increase in the chamfer angle. With the increase in the chamfer width, the cutting temperature and stress also exhibit the same variation trend.

**Figure 15:**
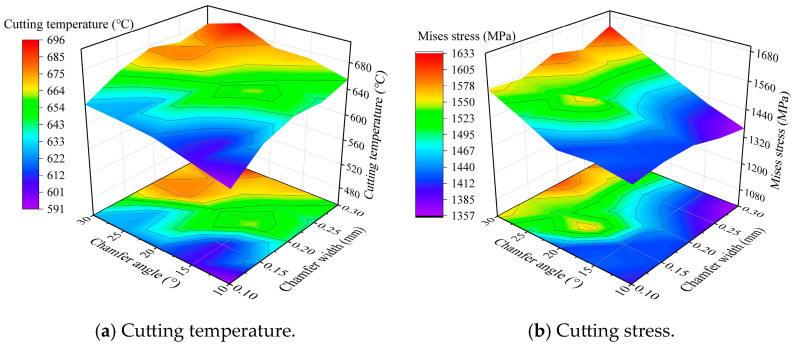
Effect of chamfer parameters on the cutting temperature and stress (*r_e_* = 30 μm).

The statistic values of the cutting temperature and stress under different chamfer parameters are depicted in Figure 15. With the increase in the chamfer width, the cutting temperature and stress exhibit a gradually increasing trend as a whole, but basically remain unchanged under the chamfer width range of 0.2~0.30 mm. With the increase in the chamfer angle, the cutting temperature and stress also exhibit a gradually increasing trend, but basically remain unchanged under the chamfer angle range of 20~30°. The highest cutting temperature and stress are obtained with the largest chamfer width and angle. There are total twenty-five combinations of the chamfer parameters with the full-factorial simulation, and there exists a combination with the smallest temperature and stress.

**Figure 16:**
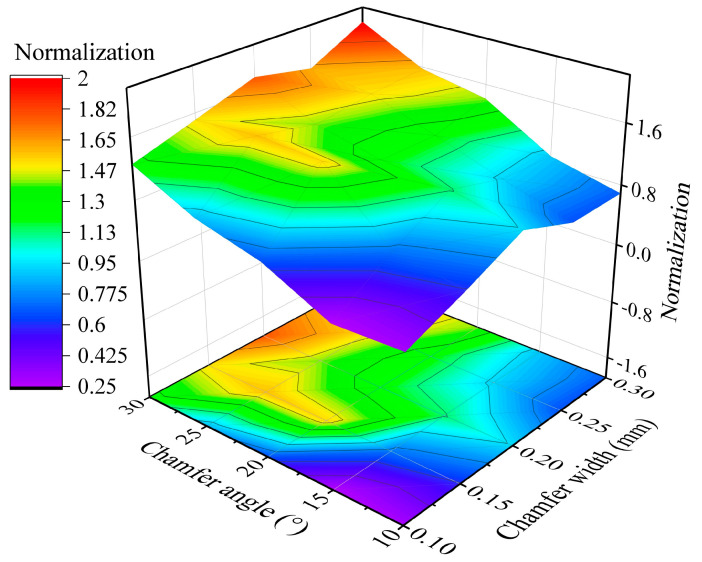
The chamfer parameters optimization based on comprehensive normalization.

Using the comprehensive normalization processing of the cutting temperature and stress in Figure 15, the calculated comprehensive normalization values are depicted in Figure 16. According to the results, the smallest comprehensive normalization value is obtained with the chamfer width of 0.1 mm and chamfer angle of 15°; simultaneously, these chamfer parameters also are consistent with one group of the preset values in the edge radius optimization. Hence, to conclude the above results, the optimal chamfer parameters of 0.1 mm/15° were achieved for the PCBN tool of the grade HNMN120712 to obtain the best tool performance.

### 4.3. The Verification of PCBN Tool in Machining of Brake Discs

**Figure 17:**
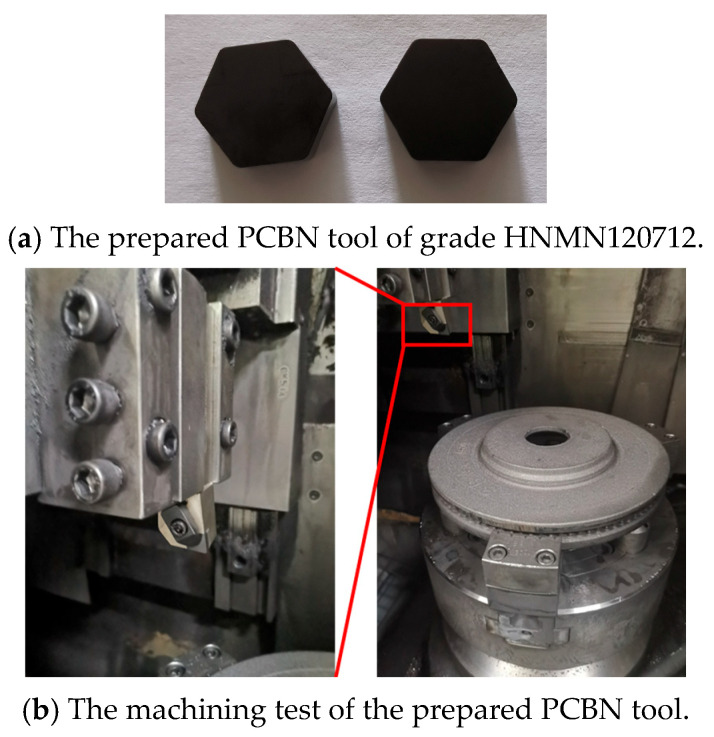
The machining test and verification of the optimized PCBN tool.

**Figure 18:**
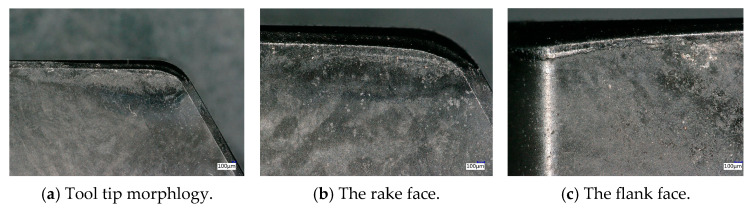
The tool wear morphology of the PCBN tool.

**Figure 19:**
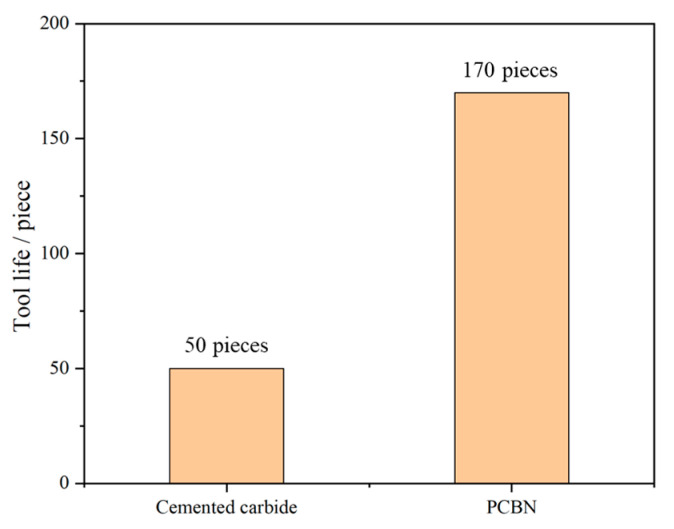
Tool life comparison of the PCBN tool and conventional tool.

After edge geometric optimization, the PCBN tool of grade HNMN120712 was prepared by Zhejiang Xinxing Tools Co., Ltd. (Jiaxing, China), as depicted in Figure 17a. Finally, the prepared PCBN tools with the optimal edge geometric parameters were tested in the machining of brake discs with the optimal turning parameters, as depicted in Figure 17b. After machining 100 pieces of brake discs, the tool wear morphology on the rake and flank faces of the prepared PCBN tool are depicted in Figure 18. It was found that there is little crater wear on the rake face, and the main tool wear is located on the flank face. With the optimized edge geometric parameters, the abnormal wear patterns are not observed; the results indicate the excellent tool performance of the optimized PCBN tool. The tool life of the PCBN tool was evaluated by a machined roughness lower than Ra 3.2 μm. The tool life comparison of one cutting edge between the optimized PCBN tool and a conventional tool, which is the currently widely used cutting tool for brake discs, is depicted in Figure 19. The designed PCBN tool was used for the actual production to replace the conventional cemented carbide tools. Hence, the prepared PCBN tool was compared to the cemented carbide tools. From the results, the tool life increases from 50 workpieces of the conventional tool to 170 workpieces of the optimized PCBN tool for each edge, which exhibits an increase ratio of 3.4 times. The tool life also indicates the excellent tool performance of the optimized PCBN tool.

## 5. Summary and Conclusions

This paper conducted an investigation on the cutting edge geometric optimization of the PCBN tool with grade HNMN120712 for the machining of brake discs. Based on the research results, the following conclusions were obtained:

1. The cutting simulation for cast iron F220P with the PCBN tool of grade HNMN120712 was established based on the P-L constitutive model. The established cutting simulation exhibits the largest error of less than 10.7% with the experiment verification. With the equal material removal rate method and considering the production efficiency requirement, the optimal turning parameters were obtained as a cutting depth of 1 mm, feed rate of 0.5 mm/r, and cutting velocity of 500 m/min.
2. The edge geometric parameters mainly include the edge radius and chamfer width and angle. The edge geometric parameters were comprehensively optimized in two stages with the normalization coefficient of the cutting temperature and stress. Firstly, the edge radius was optimized to 30 μm, and then, the chamfer width and angle were further optimized to 0.1 mm and 15°.
3. The optimized PCBN tool was prepared and then tested in the machining of brake discs made of cast iron F220P. The machining results indicate that the designed PCBN tool exhibits an excellent wear resistance performance and achieves 3.4 times the tool life of the conventional tool.

## Figures and Tables

**Table 1:** Mechanical properties of cast iron FC220P.

Parameters	Density	Tensile Strength	Yield Strength	Hardness
Value	7.1 g/cm^3^	220 MPa	180 MPa	175 HV

**Table 2:** Turning experiment results for cast iron.

Parameters	Value				
Cutting depth *a_p_*/mm	1				
Feed rate *f*/mm/r	0.4	0.45	0.5	0.55	0.6
Cutting velocity *v*/m/min	625	555	500	454	416

**Table 3:** Edge geometric parameters in the simulation.

Parameters	Value
Edge radius *r_e_*/μm	10, 20, 30, 40, 50
Chamfer angle γ/°	10, 15, 20, 25, 30
Chamfer width *b*/mm	0.1, 0.15, 0.2, 0.25, 0.3

## Data Availability

The data presented in this study are available on request from the corresponding author due to business privacy restrictions.
